# Comprehensive expression analysis of hormone-like substances in the subcutaneous adipose tissue of the common bottlenose dolphin *Tursiops truncatus*

**DOI:** 10.1038/s41598-024-63018-7

**Published:** 2024-05-31

**Authors:** Miwa Suzuki, Noriko Funasaka, Kazuma Yoshimura, Daiki Inamori, Yurie Watanabe, Miki Ozaki, Masayuki Hosono, Hideaki Shindo, Keiko Kawamura, Toshiyuki Tatsukawa, Motoi Yoshioka

**Affiliations:** 1 College of Bioresource Sciences, Nihon University, Fujisawa, Kanagawa 252-0880 Japan; 2 Cetacean Research Center, Graduate School of Bioresources, Mie University, Tsu, Mie 514-8507 Japan; 3 Taiji Whale Museum, Higashimuro, Wakayama 649-5171 Japan; 4 Adventure World, Nishimuro, Wakayama 649-2201 Japan; 5 Shimonoseki Marine Science Museum, Shimonoseki, Yamaguchi 750-0036 Japan

**Keywords:** Metabolism, Molecular biology, Physiology, Endocrinology

## Abstract

Marine mammals possess a specific subcutaneous fat layer called blubber that not only insulates and stores energy but also secretes bioactive substances. However, our understanding of its role as a secretory organ in cetaceans is incomplete. To exhaustively explore the hormone-like substances produced in dolphin subcutaneous adipose tissue, we performed seasonal blubber biopsies from captive female common bottlenose dolphins (*Tursiops truncatus;* N = 8, n = 32) and analyzed gene expression via transcriptomics. Analysis of 186 hormone-like substances revealed the expression of 58 substances involved in regulating energy metabolism, tissue growth/differentiation, vascular regulation, immunity, and ion/mineral homeostasis. Adiponectin was the most abundantly expressed gene, followed by angiopoietin protein like 4 and insulin-like growth factor 2. To investigate the endocrine/secretory responses of subcutaneous adipose tissue to the surrounding temperature, we subsequently compared the mean expression levels of the genes during the colder and warmer seasons. In the colder season, molecules associated with appetite suppression, vasodilation, and tissue proliferation were relatively highly expressed. In contrast, warmer seasons enhanced the expression of substances involved in tissue remodeling, immunity, metabolism, and vasoconstriction. These findings suggest that dolphin blubber may function as an active secretory organ involved in the regulation of metabolism, appetite, and tissue reorganization in response to changes in the surrounding environment, providing a basis for elucidating the function of hormone-like substances in group-specific evolved subcutaneous adipose tissue.

## Introduction

Adipose tissue, which is primarily composed of adipocytes laden with triglycerides, is a type of connective tissue that develops subcutaneously and surrounds or infiltrates some organs. Unlike in marine mammals, which naturally accumulate enormous amounts of fat in subcutaneous tissue, excessive fat accumulation in these tissues can induce pathological conditions in humans and laboratory animals^1^. In addition to their roles in storing energy in the form of triglycerides, retaining body heat, and protecting organs by shock absorption, adipose tissues function as secretory organs that monitor systemic energy status and produce bioactive substances called adipokines. These adipokines regulate metabolism and interact with various cells in both paracrine and endocrine manners^2,3^.

Adipokines, also referred to as adipocytokines, are low-molecular-weight proteins secreted by adipocytes. Secreted adipokines affect many tissues throughout the body, including the brain, liver, muscles, reproductive organs, and immune cells. The number of known adipokines continues to increase each year. Adiponectin (ADIPOQ), a representative adipokine, has several functions, including preventing fatty acid oxidation and inflammation, promoting blood glucose uptake by counteracting insulin resistance, and inhibiting atherosclerosis, thereby preventing obesity-related pathological states. Thus, adiponectin is often regarded as a “good” adipokine^4^. Leptin (LEP) monitors energy intake based on the amount of fat stored in fat cells and acts on the appetite center of the brain to regulate appetite and boost energy expenditure^5^. Obesity-induced changes in adipokine secretion can cause diseases such as metabolic syndrome, which is associated with increased visceral fat rather than subcutaneous fat^6^.

Cetaceans have thick subcutaneous fat that accounts for a significant portion of their weight, with reports indicating that this proportion reaches up to 28%, with a thickness of 27 mm in common bottlenose dolphins^7^. The subcutaneous adipose tissue of these animals, also called blubber, consists of two or three layers with varying adipocyte sizes and lipid compositions. While the outer layer primarily serves hydrodynamic and thermodynamic roles, the inner layer plays physiological roles in supplying and accumulating fatty acids that are mobilized for energy production (e.g.,^8,9^). Considering the diverse roles this tissue plays, in coordination with neighboring or distant tissues/cells, it is highly likely that the developed subcutaneous adipose tissue also produces and secretes various intercellular messengers, as observed in terrestrial mammals where adipose tissue functions as an endocrine and secretory organ. However, apart from studies focusing on pollutant-responsive molecules to infer the impact of marine pollutants on cetaceans (e.g.,^10^), knowledge regarding the secretory functions of their subcutaneous adipose tissue remains limited. Van Dolah et al.^10^ compared comprehensive gene expression in the skin of bottlenose dolphins in spring, summer, and winter, but no studies have tracked changes in gene expression in their subcutaneous adipose tissue in four seasons.

As an example of hormone-like substances in cetacean blubber, Ball et al.^11^ investigated the expression dynamics of LEP, its receptors, and genes related to lipid metabolism in the subcutaneous adipose tissues of bowhead whales and beluga whales living in cold regions. Their results showed that LEP expression increased in autumn compared to spring, a phenomenon likely associated with fat accumulation status. Other studies have quantified steroid hormones in the blubber to noninvasively monitor stress or reproductive status in dolphins. Their findings revealed that blubber cortisol concentration can be used as a stress indicator because it is correlated with blood levels (e.g.,^12^). Similarly, reproductive status can be ascertained by measuring blubber sex hormones (e.g.,^13,14^). Although these studies were based on the idea that hormone concentrations in the blubber reflect circulating levels, Galligan et al.^15^ revealed that enzyme-catalyzed interconversion of cortisol and cortisone occurs in the blubber of marine mammals, suggesting active steroid production in the blubber. This report highlights the role of marine mammalian subcutaneous adipose tissue as a hormone-producing organ. In a separate study, Kershaw et al.^16^ conducted a proteomic analysis of the blubber from the harbor porpoise *Phocoena phocoena*. Several proteins involved in cell function and metabolism, immune response and inflammation, and lipid metabolism were identified, but only ADIPOQ was identified as a potential hormone-like substance^16^.

An effective means to comprehensively determine the fundamental physiological roles of subcutaneous adipose tissue in cetaceans is to collect fresh samples from healthy individuals throughout the annual cycle and identify actively expressed molecules responsive to internal and external conditions using exhaustive (omics) analytical methods. In the present study, to list hormone-like substances produced in the tissue, transcriptome analysis was carried out on seasonally collected blubber biopsy samples from healthy captive common bottlenose dolphins using a minimally invasive approach. We also explored the physiological response of the subcutaneous adipose tissue to changes in the surrounding temperature by identifying hormone-like substances that exhibit altered gene expression in response to temperature fluctuations.

## Results

### Hormone-like substances expressed in the subcutaneous adipose tissue

RNA-seq analysis of 32 common dolphin subcutaneous adipose tissue samples revealed 58 hormone-like substances out of a total of 186, with median and average expression values ≥ 4 trillion per million (TPM) (Supplementary Table S1). Among these, ADIPOQ was the most abundantly expressed, with a median TPM of 1115.01. Angiopoietin protein like 4 (ANGPTL4) followed closely, along with insulin-like growth factor 2 (IGF2), which had median TPM values of 219.98 and 170.01 and constituted 21.2% and 16.3%, respectively, of the total ADIPOQ expression. Small integral membrane protein 20 (SMIM20, also known as phenoxin), complement factor D (CFD, also called adipsin), cathepsin K (CTSK), and COPI coat complex subunit alpha (COPA, also called xenin) were subsequently included in the expression hierarchy, as were the other genes. Among well-known adipokines, LEP exhibited a median TPM of 4.67, whereas resistin (RETN) did not meet the criterion of a TPM ≥ 4 (Supplementary Table  S1).

These 58 hormone-like substances, each with a TPM ≥ 4, were classified based on their generally known functions in metabolism (regulating fat and sugar metabolism in response to the body's nutritional energy status); appetite (influencing the appetite center anorexically); tissue growth/differentiation (stimulating tissue/cell growth, including angiogenesis, osteogenesis, neurogenesis, and adipocyte differentiation); generation or remodeling of blood vessels/bone/neurons; vascular control (vasodilation or vasocontraction); immunoregulation; ion/mineral homeostasis; and reproduction (Table 1). Many of these molecules have multiple functions and therefore fall into more than one category. None of the 58 genes were significantly differentially expressed according to maturity (data not shown).

**Table 1:** General categorization of hormone-like substances expressed in the subcutaneous adipose tissue of common bottlenose dolphins.

Function	Genes
Metabolism	ADIPOQ, ANGPTL4, ENHO, FBN1, IGF2, LEP, NAMPT, RARRES2
Appetite (anorexigenic)	COPA, LEP, NPFF, NUCB2, POMC, SMIM20, UCN
Tissue growth/differentiation	BMP4, HBEGF, IGF1, IGF2, SMIM20
Generation/remodeling of blood vessel/bone/neuron	*Blood vessel*: ANGPT1, ANGPT2, ANGPTL1, ANGPTL2, ANGPTL4, APLN, BMP2, CFD, NPFF, RARRES2 *Bon*e: BMP1, BMP2, BMP2K, BMP6, CSF1, CTSK, CTSL, SPP1 *Neuron*: BMP2, NTF3, OSTN,
Vascular control	*Vasoconstriction*: ACE, ADM2, EDN1 *Vasodilation*: ADM, ADM5, APLN
Immunoregulation	C3, CCL2, CSF1, CTSL, IL15, IL16, IL17C, IL33, IL34, IL36G, NAMPT, NPFF, SPP1, THPO
Ion/Mineral homeostasis	ACE, BMP6, CCL2, NUCB2, STC1, STC2, TFIP11
Reproduction	AMH, INHBB, PRLH, SMIM20
Others	APLN (*body fluid homeostasis, adipogenesis*), CTSC, CTSS (*thiol protease*), FBN1 (*tissue homeostasis*), KLK10 (*tumor suppression*), NPFF *(Pain regulation*), RBP4 (*retinol transport, insulin resistance*), SERPINE1 (*degradation of blood clots*)

Functions were extracted according to the description in the *Summaries* column of GeneCards (https://www.genecards.org/). Genes that exhibit diverse actions are classified across multiple categories.

### Changes in gene expression associated with environmental temperature

Changes in environmental temperature are likely to influence subcutaneous fat thickness and body metabolism, which, in turn, may cause changes in the production and secretion of hormone-like substances within the tissue. For the 58 substances showing a TPM ≥ 4, the RNA-seq data were divided into two groups: one from January and April, characterized by comparatively lower environmental temperatures, and the other from July and October, characterized by higher temperatures (Supplementary Figure S1). The average relative expression levels were compared between these two groups.

We found eight and 18 genes with significantly increased expression levels (*P* < 0.05) during the colder and warmer seasons, respectively (Table 2). Among the genes upregulated during colder seasons, adrenomedullin 5 (ADM5) exhibited a marked increase of 5.4-fold (adjusted *P* = 1.11e^−20^), while the others exhibited only a mild increase of less than twofold. During warmer months, secreted phosphoprotein 1 (SPP1, 7.5-fold, adjusted *P* = 2.50e^−07^), cathepsin S (CTSS, 4.1-fold, adjusted *P* = 1.37e^−13^), and retinoic acid receptor responder 2 (RARRES2, 2.4-fold, adjusted *P* = 7.62e^−11^) were upregulated > twofold compared to those in colder months, while other 15 genes were upregulated < twofold. The results for all 58 genes are presented in Supplementary Table S2.

**Table 2:** Differences in the expression of genes encoding hormone-like substances between warmer and colder seasons in the subcutaneous adipose tissue of common bottlenose dolphins.

Gene	Warmer seasons	Colder seasons	Fold change**	*P* value***
	Average ± SE*	Average ± SE		
Colder seasons > Warmer seasons				
ADM5	2.60 ± 0.23	14.10 ± 2.71	5.42	< 0.001
POMC	18.10 ± 1.88	35.60 ± 2.79	1.97	< 0.001
NPFF	4.45 ± 0.32	7.31 ± 0.69	1.64	0.010
BMP2K	10.38 ± 0.45	16.31 ± 1.08	1.57	< 0.001
SMIM20	79.84 ± 1.78	119.15 ± 4.81	1.49	< 0.001
BMP4	19.61 ± 1.52	29.04 ± 3.19	1.48	< 0.001
BMP2	8.55 ± 0.60	12.36 ± 1.20	1.45	0.002
TFIP11	17.61 ± 0.81	22.99 ± 1.20	1.31	< 0.001
Warmer seasons > Colder seasons				
SPP1	60.66 ± 41.33	8.06 ± 1.73	7.52	< 0.001
CTSS	55.96 ± 16.01	13.65 ± 1.30	4.10	< 0.001
RARRES2	71.33 ± 5.87	30.41 ± 3.37	2.35	< 0.001
INHBB	5.50 ± 0.55	2.92 ± 0.51	1.88	< 0.001
EDN1	89.70 ± 10.80	48.12 ± 6.27	1.86	< 0.001
C3	15.14 ± 3.05	8.50 ± 1.64	1.78	0.037
ANGPT1	14.41 ± 1.19	8.69 ± 1.26	1.66	< 0.001
IGF2	205.26 ± 14.73	124.89 ± 16.97	1.64	< 0.001
IL33	5.76 ± 0.63	3.64 ± 0.45	1.59	0.008
CSF1	35.91 ± 2.12	23.82 ± 2.04	1.51	< 0.001
ANGPTL1	18.00 ± 2.07	12.11 ± 1.70	1.49	0.008
NTF3	13.77 ± 1.01	9.36 ± 0.57	1.47	0.031
BMP1	31.60 ± 1.72	21.77 ± 1.48	1.45	< 0.001
FBN1	39.15 ± 3.44	27.93 ± 3.17	1.40	0.024
ANGPT2	17.17 ± 1.72	12.32 ± 1.55	1.39	0.021
ACE	31.53 ± 3.01	22.81 ± 3.09	1.38	0.046
CTSC	33.25 ± 3.48	25.05 ± 2.39	1.33	0.049
NUCB2	22.83 ± 1.64	17.53 ± 1.52	1.30	0.034

*Gene expression value is expressed as transcript per million (TPM) number.

**Fold change was determined by dividing the larger average value by the smaller value for each category.

***Differences in average were tested by paired two-sample t tests adjusted for the false discovery rate.

## Discussion

In this study, as a first step in elucidating the role of cetacean subcutaneous adipose tissue as a regulatory physiological organ, the expression of hormone-like substances in the subcutaneous adipose tissue of bottlenose dolphins was investigated at four different seasons during the year. Our findings confirmed the gene expression of 58 hormone-like substances, many of which exhibited altered expression levels in response to environmental temperature. Several studies have previously performed comprehensive molecular analyses of subcutaneous adipose tissue from cetaceans. For example, Kershaw et al.^16^ identified proteins in the blubber of harbor porpoises using proteomic techniques, including ADIPOQ and cathepsin D (CTSD). Ball et al.^11^ suggested that LEP, a hormone of which secretion is proportional to fat accumulation, is more highly expressed in the subcutaneous adipose tissue of bowhead whales, which inhabit cold regions and boast the thickest subcutaneous fat among cetaceans, than in those of other mammals. However, to the best of our knowledge, no previous reports have fully documented hormone-like substance expression in the subcutaneous adipose tissue of cetaceans across four seasons, making this study the first of its kind.

Among the commonly known adipokines^6^, ADIPOQ was particularly highly expressed in dolphin subcutaneous adipose tissue, and CFD, RARRES2, C–C motif ligand 2 (CCL2, also known as MCP-1), nicotinamide phosphoribosyl transferase (NAMPT, also called visfatin), nucleobindin 2 (NUCB2, also referred to as nesfatin 1), and CTSS were abundantly expressed. Apelin (APLN), LEP, several other cathepsins, bone morphogenetic protein (BMP) 1 and 6, lipocalin 2 (LCN2), RETN, and retinol binding protein 4 (RBP4) were expressed at relatively low levels. Other known adipokine genes described in other mammals were either expressed at lower levels or not expressed at all. In addition to the typical adipokines listed above, the expression of many other hormone-like substances has also been revealed. The physiological significance of the highly expressed or unique substances to dolphins is discussed below.

### Adiponectin

ADIPOQ was the most prominently expressed hormone-like substance in the subcutaneous adipose tissue of common bottlenose dolphins. In terrestrial mammals, ADIPOQ is actively secreted from adipocytes, with circulating levels on the order of micrograms per milliliter. It has various functions to counteract metabolic syndrome, including alleviating insulin resistance by promoting insulin-independent glucose uptake into cells and facilitating fat burning^17^. As mentioned previously, ADIPOQ has been detected in the blubber of harbor porpoises via proteomic analysis^16^, indicating the likelihood of a high protein content in the tissue. In addition, blood ADIPOQ levels in bottlenose dolphins have been reported to be comparable to those found in terrestrial mammals (e.g.,^18^). Considering the high expression of ADIPOQ in our transcriptome analysis and the aforementioned findings, it is plausible that ADIPOQ is actively produced in subcutaneous adipocytes and secreted into the blood of dolphins as in other animals^19^. Despite showing a larger encephalization quotient comparable to those of primates, dolphins have little carbohydrate in their diet and rely on gluconeogenic amino acids for endogenous glucose production, leading to insulin resistance during fasting, similar to that observed in type 2 diabetic humans, to maintain blood glucose levels^20^. Given these findings, it seems unlikely that ADIPOQ promotes cellular glucose uptake in dolphins as it does in terrestrial mammals. ADIPOQ also plays important roles in lipid metabolism, immune function, and reproduction; thus, further studies are required to clarify the physiological roles of ADIPOQ in cetaceans.

### Angiopoietin protein like 4

Following ADIPOQ, ANGPTL4 is the second most abundantly expressed hormone-like substance in dolphin subcutaneous adipose tissue. ANGPTL4 is important during fasting because it inhibits the activity of lipoprotein lipase through direct protein‒protein interactions, resulting in increased blood triglyceride levels, which are used as fuel by systemic tissues^21^. It is possible that ANGPTL4 plays a similar role in dolphins. ANGPTL4 expression is regulated in a tissue-dependent manner by nutritional (e.g., fasting) and metabolic (e.g., hypoxia) conditions. During fasting, insulin, which has an inhibitory effect on ANGPTL4, decreases, while cortisol and fatty acids, which facilitate ANGPTL4 expression, increase, suggesting that changes in these factors lead to elevated ANGPTL4 expression^22^. In the present study, blubber biopsy samples were collected in the morning following an overnight fast; therefore, the possibility of elevated ANGPTL4 expression in the tissue of dolphins must be considered. ANGPTL4 is also known to stimulate capillary network and subcutaneous adipose tissue growth through its interaction with vascular endothelial growth factors in response to PPARγ stimulation^23^. In the present study, no changes in ANGPTL4 expression were detected in response to enviromental temperature, which might affect adipose tissue thickness, suggesting that constant high expression of ANGPTL4 may support metabolic and vascular homeostasis in thick blubber; however, further studies are needed to verify this hypothesis.

### Insulin-like growth factor 2

This study revealed an interesting occurrence wherein IGF2 was highly expressed in the subcutaneous adipose tissue of dolphins. In various terrestrial mammals, IGF2 is known for its crucial function in promoting weight gain and adipocyte growth during individual development. For example, in rats, a low-protein, high-fat diet fed to mothers induces IGF2 expression in fetal adipose tissue, leading to tissue growth and obesity^24^. In human adipocytes, the local generation of IGFs is stimulated by cytokines, which contribute to the regulation of adipocyte homeostasis^25^. As our data also showed the expression of several cytokines in the blubber, a similar mechanism possibly regulating adipocyte homeostasis in dolphins; however, given its high expression, IGF2 gene is probably expressed primarily in adipocytes. In livestock, a genome-wide association analysis has highlighted the association of increased IGF2 expression, among other hormonal factors that promote adipose tissue growth, with weight gain and obesity^26^. The high expression of IGF2 in the thick blubber of the dolphin suggested that it may promote the development of adipose tissue in cetaceans, in accordance with these previous reports. In addition, the high proportion of polyunsaturated fatty acids (PUFAs) in the backfat of individuals with high IGF2-expressing genotypes suggests that IGF2 has some effect on the fatty acid composition in pigs^27^. Dolphins contain large amounts of PUFAs in their subcutaneous fat^8^, suggesting that the fatty acid composition may be regulated via IGF2.

### Small integral membrane protein 20

SMIM20 gene encodes two types of peptides that are highly conserved among mammalian species: phoenixin (PNX)-14 and PNX-20. PNX-14 is synthesized mainly in the heart and spinal cord, and PNX-20 is synthesized in the hypothalamus; however, recent evidence suggests that the PNXs produced in adipocytes promote the proliferation and differentiation of preadipocytes and are involved in white adipogenesis^28^. Other physiological effects of PNXs include appetite stimulation and suppression. Additionally, PNXs secreted from the pituitary gland and ovaries activate the female hypothalamus-pituitary–gonadal axis to stimulate LH secretion and regulate the duration of estrus^29^. Although it is reasonable to assume that the PNXs expressed in dolphin blubber are involved in the regulation of adipocytes, which also affects metabolism and reproduction, this speculation about the roles of PNXs needs to be ascertained.

### Complement factor D

CFD, also called adipsin, was also abundantly expressed in dolphin subcutaneous adipose tissue. It was one of the first adipokines to be discovered^30^. CFD is expressed in tissues during active lipid metabolism and is released into the blood. It regulates carbohydrate and lipid metabolism by controlling pancreatic β-cell function, promoting lipogenesis, and modulating inflammatory processes in response to the energy stage of the body^31^. Of the hormone-like substances found to be expressed in dolphin blubber in this study, the majority, including highly expressed molecules such as ADIPOQ, ANGPTL4, and CFD, were involved in lipid and sugar metabolism. These findings reaffirm that the dolphin subcutaneous adipose tissue functions as an important organ that monitors lipid and carbohydrate content in the body through these hormones, thereby maintaining energy balance by managing their utilization and accumulation.

### Other substances

In addition to the hormones mentioned above, many other substances known to be involved in adipocyte and adipose tissue development and differentiation were also expressed in the dolphin subcutaneous adipose tissue. These include fibrillin 1 (FBN1, also known as asprosin)^32^, cathepsins (CTSD, CTSK)^33^ and stanniocalcins (STCs)^34^, which are generally recognized for their phosphorus and calcium regulatory effects but have recently been implicated in the development, metabolism and health maintenance of adipocytes. In addition, COPA promotes fat accumulation^35^. APLN inhibits adipocyte lipolysis^36^, while NAMPT is required for adipocyte elongation in response to fat accumulation^37^. The expression of these substances likely plays a role in fat accumulation and thickening of subcutaneous adipose tissue to enhance heat retention. Energy homeostasis-associated (ENHO), which encodes adropin, inhibits lipid synthesis in white fat cells and prevents adipocyte maturation^38^. However, adropin inhibits glucose production in hepatocytes and directly promotes the use of glucose over lipids^39^, potentially contributing to fat accumulation by suppressing lipolysis. These hormone-like substances may contribute to the maintenance of adipose tissue, possibly through paracrine signaling.

Several substances related to the regulation of blood vessels are also expressed in the dolphin subcutaneous adipose tissue. Specifically, endothelin 1 (END1), ADMs, and angiotensin I-converting enzyme (ACE), which are known to be expressed in the adipose tissue of terrestrial mammals, are involved in vasoconstriction and dilation, i.e., regulation of blood pressure and blood flow (e.g.,^40,41^). In the dolphin blubber, these hormones may also regulate local perfusion of tissue in response to changes in water and air temperature, intensity of solar radiation and diving behavior. The expression of angiopoietins and APLN, which are known to be involved in angiogenesis^42^, was also detected. Like other marine mammals, dolphins have a relatively large number of capillaries in their subcutaneous adipose tissue (e.g.,^43^); thus, these substances may play a role in maintaining and regulating these vessels.

Some cytokines were expressed in the dolphin blubber. Unless single-cell RNA-seq is performed, transcriptome analysis alone cannot definitively distinguish whether the substances expressed in the subcutaneous adipose tissue of dolphins originate from adipocytes or other cell types found in the tissue. Adipose tissue is a potent source of inflammatory interleukins and other cytokines, and subcutaneous adipocytes may produce cytokines, as revealed in human adipocytes^44^. However, similar to those in humans, most cytokines are likely produced by other cell types within adipose tissue^45^. Numerous microvessels are present in dolphin blubber^43^, suggesting that cytokines may be expressed by some kinds of leukocytes in blood vessels or may migrate into tissues.

An unexpected finding in this study was the expression of anti-Müllerian tubular hormone (AMH) in the dolphin subcutaneous adipose tissue. In terrestrial mammals, AMH is avital hormone that regulates the number of reproductive cells by modulating stem cells^46^. In addition, LEP is known to negatively impact reproductive function; obesity promotes LEP secretion from adipose tissue, which in turn acts on the pituitary gland to induce the secretion of luteinizing hormone and follicle-stimulating hormone, thereby overstimulating the ovaries, reducing ovarian reserves, and impairing female reproductive function (e.g.,^47^). Furthermore, IGF2 is known to affect the ovary, promote follicle formation and granulosa cell proliferation, and maintain oocyte integrity^48^. In addition to the hormones mentioned above, several adipokines have been recognized to affect female reproductive functions by modulating fat mass and insulin sensitivity^49^. Although the effects of these gene products expressed in the dolphin blubber on the reproductive system possibly differ from their actions in terrestrial mammals and these hormones may also regulate the female reproductive system as signals of fat accumulation and metabolic status, given the amount of these hormones secreted by subcutaneous adipose tissue, which accounts for a large percentage of body weight, may reach nonnegligible levels and could affect the gonads, but this warrants careful investigation.

### Substances showing expression changes in response to environmental temperature

Among the hormone-like substances expressed in the subcutaneous adipose tissue of dolphins, several were found to change their expression levels in response to changes in water and air temperature.

In the colder seasons (January and April), the expression of proopiomelanocortin (POMC), the neuropeptide FF-amide peptide precursor (NPFF), and SMIM20 increased. These hormones regulate appetite through the central nervous system. Alpha-melanocyte-stimulating hormone derived from POMC acts as an appetite suppressor via melanocortin type 4 receptors in the hypothalamus^50^. Similarly to POMC, the NPFF also suppresses appetite functions in central appetite regulation^51^. However, the significance of enhanced POMC and NPFF production in adipocytes during the colder season for appetite regulation is unclear. POMC is also known to regulate lipolysis in adipocytes via LEP and insulin signaling and to promote the transformation to brown adipose tissue (BAT)^52^. Although the presence of brown adipocytes in cetaceans remains uncertain^53,54^, an increase in nonshivering heat-producing BAT during the colder season may be beneficial for body heat retention. PNX-14 and PNX-20 derived from SMIM20 are also involved in appetite regulation and have both stimulating and suppressing effects; however, PNXs are known to activate the female reproductive axis as discussed above^29^. These appetite-regulating hormones may be linked to appetite control while monitoring the nutritional status and/or surrounding temperature. In addition, seasonal fluctuations in circulating hormones controlling reproduction and/or metabolism, which are observed in common bottlenose dolphins^55,56^, may influence the expression of these adipokines, which affect appetites. However, further research is needed to elucidate the roles of these substances and the mechanisms that regulate their expression. The expression of ADM5, which possesses hemodynamic actions (vasodilation), including decreasing peripheral resistance^57^, was enhanced. This finding suggested that dolphin subcutaneous adipose tissue responds to coldness by allowing more blood to circulate, which may have an antagonistic effect on the vasoconstriction of skin tissue during cold weather.

In warmer seasons with higher environmental temperatures (July and October), SPP1 exhibited significantly elevated expression compared to that in colder months. SPP1 is a multifunctional hormone that is expressed in adipocyte progenitor cells and possibly inhibits adipogenesis^58^. The expression of RARRES2, which synthesizes chemerin, also increased. It suppresses appetite in an endocrine manner, induces lipolytic metabolism, and promotes adipocyte differentiation^59^. In addition, the expression of IGF2, which, as previously mentioned, can influence fatty acid composition and increase the PUFA content in pigs^27^, was similarly enhanced. These actions may lead to reduced fat mass and increased fat fluidity, potentially resulting in a relative reduction in the thermoretentive effects of subcutaneous fat during warmer seasons. Colony stimulating factor 1 (CSF1) expression also increased during the warmer season. CSF1 is a well-known regulator of macrophage survival, proliferation, and chemotaxis; however, recent reports suggest that it may play a role in regulating lipid metabolism in adipocytes^60^, suggesting its possible contribution to changes in adipocyte lipid metabolism in response to environmental temperature.

In addition, the expression of ANGPT1, which has vasoprotective and angiogenic effects that promote vascular survival, inhibit leakage, and reduce inflammation^61^, was also increased during the warmer season, in addition to other substances involved in vascular remodeling. The expression of EDN1 and ACE, both of which constrict blood vessels^40,62^, also increased. Considering that the expression of vasodilators conversely increased during the colder season, it is assumed that vasodilation, contraction, and structural stability of the blood vessels within the tissue are associated with vascular remodeling functioning to control heat loss from the dermis during warmer and colder seasons and/or fluctuations in subcutaneous fat volume.

Inhibin subunit beta B (INHBB) is a component of activin or inhibin and is well known for its ability to regulate reproduction and development; however, it is also produced in the form of activin B in rodent adipocytes and is suggested to be involved in the regulation of energy balance under the control of LEP^63^. The expression of NUCB2, known for its appetite-suppressing properties^64^, also increased during the warmer season. NUCB2 expressed in adipocytes has been reported to influence gonad maturation and enhance function through energy homeostasis^65^. The physiological significance of the increased expression of these hormones during warmer months, when dolphin reproductive activity is relatively high, requires further study.

### Limitations

As the first step toward elucidating the role of dolphin subcutaneous adipose tissue as an endocrine organ, we aimed to comprehensively characterize the substances expressed in the tissue based on transcriptome data obtained from eight captive female common bottlenose dolphins across four seasons. Although the data revealed valuable insights, the strength and importance of the action of each substance cannot be assessed based solely on gene expression levels. To gain a more in-depth understanding, it is necessary to analyze the translation of these genes into proteins and their secretion from adipose tissue, whether they function in an endocrine or paracrine manner, and the distribution of their receptors, as well as their effects on target cells. In addition, the sex ratio of captive common bottlenose dolphins is biased toward females, making it difficult to conduct experiments with a sufficient number of mature and immature males kept in similar environments; therefore, only females were included in this study. However, studies on males are necessary to elucidate the expression dynamics and function of hormone-like substances in the blubber.

In northern elephant seals, the secretion patterns of four adipokines at various stages of life history were scrutinized, and the results suggested that the expression and function of adipose-derived hormones in species that undergo natural significant changes in body fat percentage as part of their life history may differ in some respects from those in humans and model animals^66^. Since dolphins do not have a life history stage characterized by a marked increase or decrease in body fat accumulation observed in the elephant seal, it is unlikely that the same functional changes in adipokines occur in dolphins as in the seal. However, dolphins, like seals, naturally accumulate large amounts of fat, and their glucose utilization methods are different from those of humans and model animals^20^; furthermore, changes in the amount and composition of subcutaneous fat to some extent are observed in response to changes in environmental temperature and the presence or absence of lactation^67^. Therefore, as reiterated in the discussion, it should be noted that the functions of each adipokine discovered in terrestrial mammals may not apply to dolphins and require careful future studies.

## Conclusion

Using year-round blubber biopsy samples and transcriptome analysis techniques, this study revealed that a variety of peptide/protein hormones other than the well-known adipokines secreted from adipose tissues in terrestrial mammals are expressed in dolphin subcutaneous adipose tissue. The expression levels of hormones involved in adipocyte growth and maturation, vascular regulation, reproduction and other processes vary seasonally. While some hormones may act in a paracrine manner to maintain the thick subcutaneous fat of dolphins, others, especially those with high expression, may act in an endocrine manner to elicit systemic responses. Cetaceans are the most successfully adapted mammals to the ocean of any group, and they evolved to accumulate vast amounts of fat in the blubber. Unlike humans and model animals, high obesity is their natural state, and as a consequence, at least some of the endocrine functions of their subcutaneous adipose tissue may have been modified/evolved in a group-specific manner. The gene expression patterns of hormone-like substances in the blubber of dolphins provide a basis for investigating these characteristics. Our findings have great implications for understanding the physiological role of subcutaneous adipose tissue in maintaining homeostasis in cetaceans, a valuable evolutionary model organism group of oceanic adaptation. In addition, comparison of the gene expression patterns with those of humans and other model animals will help to elucidate the reasons why highly obese individuals do not develop pathological conditions in marine mammals.

## Methods

### Experimental setup

This study involved eight healthy female common bottlenose dolphins, *Tursiops truncatus*, i.e., one immature and one mature dolphins from the Shimonoseki Marine Science Museum, Yamaguchi, Japan; two matures from the Adventure World, Wakayama, Japan; and three immature individuals and one dolphin that was possibly matured from immature during the experimental period from the Taiji Whale Museum, Wakayama, Japan (Supplementary Table S3). The annual changes in air temperature (maximum and minimum) and water temperature at each aquarium are shown in Supplementary Figure S1. At Shimonoseki, the water temperature in the tank was controlled to avoid temperatures below 18 °C and above 24 °C; otherwise, the dolphins were kept at natural ambient temperatures that showed similar temperature changes among the aquaria. The maturity of the dolphins was determined based on the basal levels of circulating progesterone and the presence or absence of an increase in the concentration of progesterone (data not shown). This study complied with the ARRIVE guidelines. All experiments were conducted in compliance with the guidelines for animal experiments at Nihon University, and the experimental protocols were approved by Nihon University (approval number: AP19BRS110-2).

### Biopsy sampling

Blubber biopsy was performed in April, July, and October 2021 and January 2022 following the methods of Funasaka et al.^68^. In brief, before the start of the day's feeding, the dolphins were administered sedatives and were locally anesthetized in the muscle before sampling when necessary. After the skin surface was sterilized with alcohol, biopsies were carried out using a sterilized puncher (Shiba-puncher, SP-203, SSS-System, Kyoto, Japan; Supplementary Figure S2). The collected specimens, approximately 3 mm in diameter and 30 mm in length, were immediately frozen in liquid nitrogen after sampling and stored at − 80 °C until analysis. The health status and wound healing process of the dolphins were monitored three weeks after the experiment using blood biochemistry tests and external observation of the wound appearance, with minimal impact on their health observed^68^.

### RNA sequencing and expression analyses

RNA sequencing was carried out following the methods of Suzuki et al.^69^. The outer layers of the skin (epidermis) and muscle were swiftly removed from the biopsy specimens on dry ice. The whole blubber tissue was then thawed, and all of the tissue was promptly homogenized in Isogen (Nippon Gene, Tokyo, Japan) at room temperature (20–25 °C). Total RNA was extracted according to the manufacturer’s instructions. The RNA integrity of the extracted total RNA ranged from 5.9 to 7.1 (average 6.4 ± 0.1). RNA-seq was conducted using an Illumina NovaSeq platform (Illumina, CA, USA) in a 150 bp paired-end configuration. The resulting sequences were analyzed using the transcriptomic analysis platform Qiagen CLC Genomic Workbench 12.0 (Qiagen, Aarhus, Denmark)^70^. Briefly, after the removal of adaptor sequences and trimming of low-quality sequences (Q score < 30), the resultant high-quality reads were mapped to a reference genome (GCF_011762595.1_mTurTru1.mat._Y_genomic, https://www.ncbi.nlm.nih.gov/datasets/genome/GCF_011762595.1/). The calculated TPM value was used for expression analysis of each gene.

Following Wagner et al.^71^, we adopted TPM ≥ 4 for both the median and average values as cutoff criteria to determine whether a gene was actively expressed among the 186 hormone-like substances (listed in Supplementary Table S1), including representative adipokines^6,72^. Hormone-like substances not annotated in the reference genome of common bottlenose dolphin were excluded from the analysis.

For substances with TPM ≥ 4, we conducted a paired two-sample t test adjusted for the false discovery rate using the Benjamini–Hochberg method to evaluate the difference in average gene expression levels between July and October, which had relatively high environmental temperatures, and January and April, which had lower temperatures (Supplementary Figure S1). The same test was also conducted to examine the difference between immature and mature dolphins, in which an individual who was judged to have reached maturity based on the pattern of fluctuations in blood progesterone (data not shown) during the experiment was excluded from the analysis because her maturity status did not fit into either category. The significance level was set at an adjusted *P* value < 0.05.

### Clarification

Midazolam and butorphanol were used for sedation of dolphins, and lidocaine hydrochloride was administered for local anesthesia at an adjusted dose for individuals under the direction of veterinarians. Nihon University has an ethics committee that reviews the appropriateness of animal experiments in accordance with the guidelines for animal experiments at Nihon University.

### Supplementary Information

Supplementary Figures.

Supplementary Tables.

## Data Availability

RNA sequencing (RNA-seq) data are available in the DDBJ Sequence Read Archive (https://ddbj.nig.ac.jp/search; accession numbers DRR513289-DRR513320).
