# Supplementary material for: Comprehensive expression analysis of hormone-like substances in the subcutaneous adipose tissue of the common bottlenose dolphin Tursiops truncatus
Source: Sci Rep. 2024 May 31;14:12515. doi: 10.1038/s41598-024-63018-7 (PMC11143283; doi:10.1038/s41598-024-63018-7)
Supplement: Supplementary file 1 — Supplementary Figures. [file 41598_2024_63018_MOESM1_ESM.pdf]

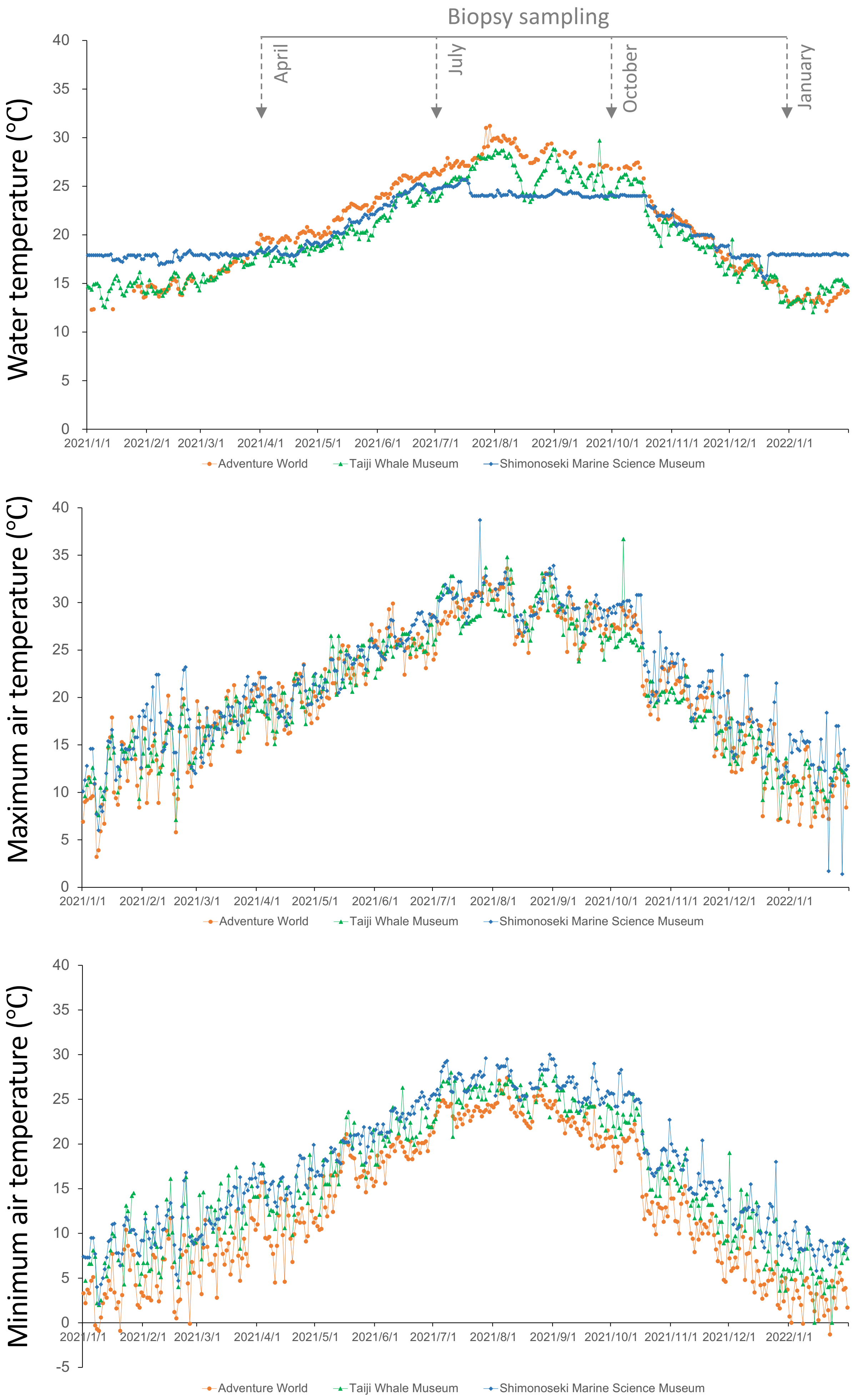

Supplementary Figure S1. Annual changes in water temperature in the tank (upper panel), maximum water temperature (middle panel) and minimum water temperature (bottom panel) in each aquarium from January 2021 to January 2022. The timing of the blubber biopsy sampling is shown at the top.

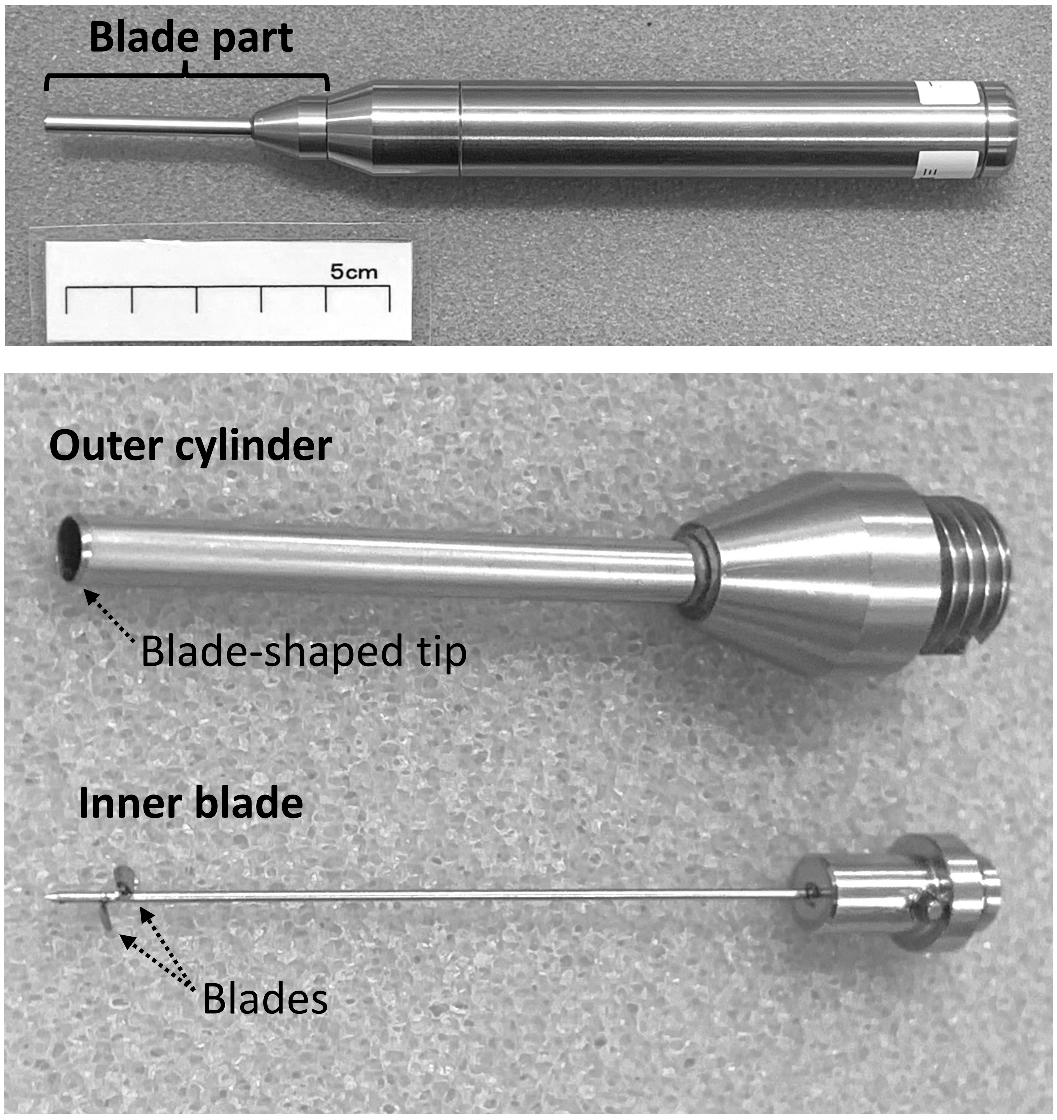

Supplementary Figure S2. The puncher used for blubber biopsy collection from dolphins. The upper picture shows an overall view, and the lower picture shows a magnified view of the apex part consisting of an outer syringe and an inside core with two feather-shaped blades. A blubber sample is collected by puncturing while rotating the puncher.
