# Supplementary material for: Comprehensive expression analysis of hormone-like substances in the subcutaneous adipose tissue of the common bottlenose dolphin Tursiops truncatus
Source: Sci Rep. 2024 May 31;14:12515. doi: 10.1038/s41598-024-63018-7 (PMC11143283; doi:10.1038/s41598-024-63018-7)
Supplement: Supplementary file 2 — Supplementary Tables. [file 41598_2024_63018_MOESM2_ESM.docx]

**Supplementary Table S1**. Genes of hormone-like substances expressed in the subcutaneous adipose tissue of common bottlenose dolphins showing median and average with standard error of transcript per million (TPM) number. Genes are sorted by the median TPM value.

|  | Gene Name | TPM | | |
| --- | --- | --- | --- | --- |
|  |  | Median | Average | SE |
| 1 | ADIPOQ | 1115.01 | 1187.92 | 156.46 |
| 2 | ANGPTL4 | 219.98 | 242.35 | 23.93 |
| 3 | IGF2 | 170.01 | 164.36 | 13.42 |
| 4 | SMIM20 | 99.51 | 100.92 | 4.34 |
| 5 | CFD | 93.43 | 109.89 | 13.85 |
| 6 | CTSK | 91.99 | 91.94 | 3.09 |
| 7 | COPA | 78.69 | 77.85 | 2.33 |
| 8 | EDN1 | 59.78 | 68.75 | 7.25 |
| 9 | ANGPTL2 | 54.55 | 58.33 | 4.56 |
| 10 | KLK10 | 49.39 | 97.15 | 21.40 |
| 11 | CCL2 | 46.36 | 57.69 | 6.21 |
| 12 | BMP6 | 43.51 | 42.71 | 3.02 |
| 13 | RARRES2 | 41.87 | 49.70 | 4.99 |
| 14 | SERPINE1 | 34.88 | 40.41 | 3.05 |
| 15 | ENHO | 33.67 | 34.93 | 3.44 |
| 16 | FBN1 | 31.92 | 33.60 | 2.54 |
| 17 | CSF1 | 30.65 | 29.76 | 1.83 |
| 18 | POMC | 27.59 | 27.61 | 2.27 |
| 19 | CTSC | 27.53 | 29.10 | 2.23 |
| 20 | ACE | 26.59 | 27.41 | 2.27 |
| 21 | BMP1 | 26.03 | 26.41 | 1.44 |
| 22 | CTSS | 23.17 | 34.77 | 8.77 |
| 23 | UCN | 23.13 | 22.61 | 1.06 |
| 24 | RBP4 | 22.91 | 23.48 | 2.13 |
| 25 | NAMPT | 22.87 | 24.75 | 1.65 |
| 26 | BMP4 | 22.28 | 24.66 | 1.97 |
| 27 | NUCB2 | 19.81 | 20.10 | 1.22 |
| 28 | TFIP11 | 19.79 | 20.66 | 0.83 |
| 29 | IL16 | 17.56 | 18.50 | 1.37 |
| 30 | ANGPTL1 | 14.30 | 14.87 | 1.44 |
| 31 | ANGPT2 | 13.85 | 14.73 | 1.23 |
| 32 | ADM | 12.85 | 14.18 | 1.00 |
| 33 | IL34 | 12.23 | 12.52 | 0.55 |
| 34 | BMP2K | 11.90 | 13.47 | 0.80 |
| 35 | AMH | 11.46 | 12.43 | 0.93 |
| 36 | HBEGF | 11.29 | 11.31 | 0.52 |
| 37 | NTF3 | 11.08 | 11.67 | 0.68 |
| 38 | ANGPT1 | 10.85 | 11.14 | 0.95 |
| 39 | APLN | 10.49 | 12.07 | 1.08 |
| 40 | BMP2 | 10.36 | 10.66 | 0.74 |
| 41 | SPP1 | 9.78 | 34.45 | 20.88 |
| 42 | CTSL | 8.97 | 10.16 | 0.76 |
| 43 | C3 | 8.45 | 11.83 | 1.81 |
| 44 | OSTN | 8.21 | 8.65 | 0.78 |
| 45 | ADM2 | 7.53 | 7.61 | 0.65 |
| 46 | STC1 | 6.94 | 9.20 | 1.12 |
| 47 | IL36G | 5.90 | 38.41 | 18.04 |
| 48 | PRLH | 5.38 | 7.52 | 1.11 |
| 49 | IGF1 | 5.34 | 6.03 | 0.52 |
| 50 | IL15 | 5.23 | 5.29 | 0.43 |
| 51 | ADM5 | 5.23 | 8.70 | 1.73 |
| 52 | NPFF | 5.09 | 5.95 | 0.46 |
| 53 | STC2 | 4.79 | 5.15 | 0.38 |
| 54 | THPO | 4.69 | 4.74 | 0.29 |
| 55 | LEP | 4.67 | 6.56 | 1.22 |
| 56 | IL17C | 4.48 | 5.57 | 0.62 |
| 57 | INHBB | 4.07 | 4.17 | 0.44 |
| 58 | IL33 | 4.04 | 4.60 | 0.43 |
| 59 | HCRT | 3.98 | 4.00 | 0.46 |
| 60 | INHBA | 3.62 | 3.89 | 0.36 |
| 61 | NPPC | 3.49 | 3.88 | 0.45 |
| 62 | PDYN | 3.11 | 3.99 | 0.56 |
| 63 | FST | 3.07 | 3.60 | 0.42 |
| 64 | ANGPTL7 | 2.74 | 3.25 | 0.44 |
| 65 | IL18 | 2.63 | 2.50 | 0.23 |
| 66 | LCN2 | 2.55 | 3.87 | 0.63 |
| 67 | IL17B | 2.45 | 2.89 | 0.23 |
| 68 | CSF3 | 2.08 | 2.36 | 0.25 |
| 69 | RETN | 2.00 | 2.21 | 0.22 |
| 70 | PTHLH | 1.97 | 2.57 | 0.33 |
| 71 | PTH | 1.91 | 2.05 | 0.18 |
| 72 | GAL | 1.75 | 1.88 | 0.24 |
| 73 | IL17D | 1.61 | 1.76 | 0.11 |
| 74 | ACE2 | 1.59 | 1.76 | 0.14 |
| 75 | INHA | 1.52 | 1.52 | 0.12 |
| 76 | IL12A | 1.45 | 1.97 | 0.27 |
| 77 | BMP7 | 1.41 | 1.90 | 0.40 |
| 78 | IL11 | 1.12 | 1.09 | 0.11 |
| 79 | IL1A | 1.11 | 1.97 | 0.61 |
| 80 | ANGPT4 | 1.09 | 1.92 | 0.33 |
| 81 | ANGPTL5 | 1.01 | 1.30 | 0.21 |
| 82 | ASIP | 0.83 | 0.92 | 0.09 |
| 83 | IL7 | 0.78 | 1.03 | 0.10 |
| 84 | NGF | 0.76 | 0.88 | 0.09 |
| 85 | IL6 | 0.72 | 0.86 | 0.10 |
| 86 | TAC3 | 0.69 | 0.89 | 0.14 |
| 87 | AGRP | 0.68 | 0.76 | 0.09 |
| 88 | MZB1 | 0.64 | 0.78 | 0.12 |
| 89 | TRH | 0.61 | 1.04 | 0.19 |
| 90 | IL23A | 0.53 | 0.53 | 0.04 |
| 91 | EDN2 | 0.52 | 0.61 | 0.09 |
| 92 | NMU | 0.50 | 0.71 | 0.09 |
| 93 | ANGPTL6 | 0.47 | 0.61 | 0.06 |
| 94 | BMP5 | 0.45 | 0.57 | 0.07 |
| 95 | IL17F | 0.39 | 0.67 | 0.12 |
| 96 | NPW | 0.39 | 0.41 | 0.07 |
| 97 | GHRL | 0.38 | 0.50 | 0.09 |
| 98 | IL1B | 0.37 | 0.63 | 0.14 |
| 99 | KLK14 | 0.35 | 0.90 | 0.30 |
| 100 | HAMP | 0.33 | 0.88 | 0.31 |
| 101 | AGT | 0.30 | 0.37 | 0.06 |
| 102 | NPY | 0.22 | 0.68 | 0.19 |
| 103 | OXT | 0.22 | 0.43 | 0.08 |
| 104 | IL10 | 0.22 | 0.30 | 0.05 |
| 105 | TNF | 0.18 | 0.32 | 0.09 |
| 106 | KLK12 | 0.17 | 0.26 | 0.06 |
| 107 | APELA | 0.15 | 0.24 | 0.06 |
| 108 | IL12B | 0.14 | 0.17 | 0.03 |
| 109 | IFNE | 0.13 | 0.14 | 0.02 |
| 110 | NTS | 0.11 | 0.26 | 0.07 |
| 111 | BDNF | 0.11 | 0.16 | 0.03 |
| 112 | PENK | 0.10 | 0.25 | 0.06 |
| 113 | ANGPTL3 | 0.10 | 0.31 | 0.10 |
| 114 | CCK | 0.08 | 0.41 | 0.13 |
| 115 | VIP | 0.07 | 0.14 | 0.03 |
| 116 | REN | 0.07 | 0.12 | 0.03 |
| 117 | KNG1 | 0.05 | 0.33 | 0.18 |
| 118 | IL27 | 0.05 | 0.27 | 0.08 |
| 119 | TAFA5 | 0.04 | 0.08 | 0.02 |
| 120 | SST | 0.02 | 0.15 | 0.05 |
| 121 | PRL | 0.00 | 1.52 | 0.94 |
| 122 | ANGPTL8 | 0.00 | 0.30 | 0.09 |
| 123 | LCN8 | 0.00 | 0.21 | 0.07 |
| 124 | IL19 | 0.00 | 0.13 | 0.04 |
| 125 | GCG | 0.00 | 0.12 | 0.03 |
| 126 | GAST | 0.00 | 0.12 | 0.08 |
| 127 | KLK5 | 0.00 | 0.11 | 0.03 |
| 128 | CRSP1-like | 0.00 | 0.11 | 0.04 |
| 129 | prorelaxin | 0.00 | 0.11 | 0.03 |
| 130 | IL5 | 0.00 | 0.10 | 0.04 |
| 131 | IL36B | 0.00 | 0.10 | 0.05 |
| 132 | CALCB | 0.00 | 0.10 | 0.03 |
| 133 | FGF23 | 0.00 | 0.09 | 0.04 |
| 134 | INS | 0.00 | 0.08 | 0.03 |
| 135 | CRSP2-like | 0.00 | 0.08 | 0.03 |
| 136 | LCN6 | 0.00 | 0.08 | 0.03 |
| 137 | TSHB | 0.00 | 0.07 | 0.04 |
| 138 | PMCH | 0.00 | 0.07 | 0.04 |
| 139 | EPO | 0.00 | 0.07 | 0.02 |
| 140 | VGF | 0.00 | 0.06 | 0.02 |
| 141 | CRH | 0.00 | 0.06 | 0.02 |
| 142 | GRP | 0.00 | 0.06 | 0.03 |
| 143 | KLK1 | 0.00 | 0.04 | 0.02 |
| 144 | PNOC | 0.00 | 0.04 | 0.02 |
| 145 | BMP3 | 0.00 | 0.03 | 0.02 |
| 146 | UTS2 | 0.00 | 0.03 | 0.02 |
| 147 | ADCYAP1 | 0.00 | 0.03 | 0.01 |
| 148 | CSF2 | 0.00 | 0.03 | 0.01 |
| 149 | IFNG | 0.00 | 0.03 | 0.03 |
| 150 | FNDC5 | 0.00 | 0.03 | 0.01 |
| 151 | TAC1 | 0.00 | 0.02 | 0.01 |
| 152 | GHRH | 0.00 | 0.02 | 0.02 |
| 153 | NPVF | 0.00 | 0.02 | 0.01 |
| 154 | IL24 | 0.00 | 0.02 | 0.01 |
| 155 | IL20 | 0.00 | 0.02 | 0.01 |
| 156 | INHBE | 0.00 | 0.02 | 0.01 |
| 157 | EDN3 | 0.00 | 0.01 | 0.01 |
| 158 | FSHB | 0.00 | 0.01 | 0.01 |
| 159 | NPPB | 0.00 | 0.01 | 0.01 |
| 160 | IL13 | 0.00 | 0.01 | 0.01 |
| 161 | FGF21 | 0.00 | 0.01 | 0.00 |
| 162 | AVP | 0.00 | 0.01 | 0.01 |
| 163 | IFNK | 0.00 | 0.01 | 0.01 |
| 164 | CARTPT | 0.00 | 0.00 | 0.00 |
| 165 | BMP10 | 0.00 | 0.00 | 0.00 |
| 166 | BMP15 | 0.00 | 0.00 | 0.00 |
| 167 | GIP | 0.00 | 0.00 | 0.00 |
| 168 | GNRH1 | 0.00 | 0.00 | 0.00 |
| 169 | GUCY1B2-like | 0.00 | 0.00 | 0.00 |
| 170 | IFNB1 | 0.00 | 0.00 | 0.00 |
| 171 | IFNL3 | 0.00 | 0.00 | 0.00 |
| 172 | IL17A | 0.00 | 0.00 | 0.00 |
| 173 | IL2 | 0.00 | 0.00 | 0.00 |
| 174 | IL21 | 0.00 | 0.00 | 0.00 |
| 175 | IL22 | 0.00 | 0.00 | 0.00 |
| 176 | IL26 | 0.00 | 0.00 | 0.00 |
| 177 | IL4 | 0.00 | 0.00 | 0.00 |
| 178 | IL9 | 0.00 | 0.00 | 0.00 |
| 179 | INHBC | 0.00 | 0.00 | 0.00 |
| 180 | ITLN1 | 0.00 | 0.00 | 0.00 |
| 181 | LCN10 | 0.00 | 0.00 | 0.00 |
| 182 | LCN15 | 0.00 | 0.00 | 0.00 |
| 183 | MLN | 0.00 | 0.00 | 0.00 |
| 184 | NPPA | 0.00 | 0.00 | 0.00 |
| 185 | NPS | 0.00 | 0.00 | 0.00 |
| 186 | PPY | 0.00 | 0.00 | 0.00 |

**Supplementary** **Table S2**. Differences in averages of transcript per million (TPM) number between warmer and colder seasons for 58 genes expressed in the subcutaneous adipose tissue of common bottlenose dolphin (n=32) with TPM value ≥ 4. Genes are sorted by the median TPM value.

|  | Gene Name | TPM (Warmer) | | TPM (Colder) | | Warmer season vs colder season | |
| --- | --- | --- | --- | --- | --- | --- | --- |
|  |  | Average | SE | Average | SE | Fold change | p-value* |
| 1 | ADIPOQ | 1152.45 | 144.44 | 1287.44 | 281.95 | -1.12 | 0.998 |
| 2 | ANGPTL4 | 259.38 | 30.64 | 223.67 | 36.13 | 1.16 | 0.485 |
| 3 | IGF2 | 205.26 | 14.73 | 124.89 | 16.97 | 1.64 | **<0.001** |
| 4 | SMIM20 | 79.84 | 1.78 | 119.15 | 4.81 | -1.49 | **<0.001** |
| 5 | CFD | 105.75 | 26.30 | 111.14 | 10.09 | -1.05 | 0.695 |
| 6 | CTSK | 97.49 | 4.05 | 85.61 | 4.29 | 1.14 | 0.253 |
| 7 | COPA | 80.62 | 3.20 | 74.57 | 3.28 | 1.08 | 0.515 |
| 8 | EDN1 | 89.70 | 10.80 | 48.12 | 6.27 | 1.86 | **<0.001** |
| 9 | ANGPTL2 | 58.47 | 3.98 | 57.97 | 8.11 | 1.01 | 0.902 |
| 10 | KLK10 | 73.49 | 24.42 | 131.87 | 35.70 | -1.79 | 0.248 |
| 11 | CCL2 | 52.53 | 7.94 | 61.21 | 9.49 | -1.17 | 0.498 |
| 12 | BMP6 | 42.99 | 3.55 | 42.19 | 4.86 | 1.02 | 0.929 |
| 13 | RARRES2 | 71.33 | 5.87 | 30.41 | 3.37 | 2.35 | **<0.001** |
| 14 | SERPINE1 | 42.83 | 4.00 | 37.34 | 4.55 | 1.15 | 0.557 |
| 15 | ENHO | 28.42 | 2.69 | 41.44 | 5.80 | -1.46 | 0.067 |
| 16 | FBN1 | 39.15 | 3.44 | 27.93 | 3.17 | 1.40 | **0.024** |
| 17 | CSF1 | 35.91 | 2.12 | 23.82 | 2.04 | 1.51 | **<0.001** |
| 18 | POMC | 18.10 | 1.88 | 35.60 | 2.79 | -1.97 | **<0.001** |
| 19 | CTSC | 33.25 | 3.48 | 25.05 | 2.39 | 1.33 | **0.049** |
| 20 | ACE | 31.53 | 3.01 | 22.81 | 3.09 | 1.38 | **0.046** |
| 21 | BMP1 | 31.60 | 1.72 | 21.77 | 1.48 | 1.45 | **<0.001** |
| 22 | CTSS | 55.96 | 16.01 | 13.65 | 1.30 | 4.10 | **<0.001** |
| 23 | UCN | 23.42 | 1.38 | 21.26 | 1.68 | 1.10 | 0.805 |
| 24 | RBP4 | 26.25 | 3.58 | 22.51 | 2.84 | 1.17 | 0.299 |
| 25 | NAMPT | 22.87 | 1.46 | 26.77 | 2.85 | -1.17 | 0.305 |
| 26 | BMP4 | 19.61 | 1.52 | 29.04 | 3.19 | -1.48 | **<0.001** |
| 27 | NUCB2 | 22.83 | 1.64 | 17.53 | 1.52 | 1.30 | **0.034** |
| 28 | TFIP11 | 17.61 | 0.81 | 22.99 | 1.20 | -1.31 | **<0.001** |
| 29 | IL16 | 17.56 | 1.15 | 18.83 | 2.51 | -1.07 | 0.568 |
| 30 | ANGPTL1 | 18.00 | 2.07 | 12.11 | 1.70 | 1.49 | **0.008** |
| 31 | ANGPT2 | 17.17 | 1.72 | 12.32 | 1.55 | 1.39 | **0.021** |
| 32 | ADM | 15.13 | 1.19 | 13.64 | 1.63 | 1.11 | 0.417 |
| 33 | IL34 | 11.48 | 0.69 | 13.24 | 0.83 | -1.15 | 0.090 |
| 34 | BMP2K | 10.38 | 0.45 | 16.31 | 1.08 | -1.57 | **<0.001** |
| 35 | AMH | 12.62 | 1.41 | 11.91 | 1.26 | 1.06 | 0.995 |
| 36 | HBEGF | 12.27 | 0.72 | 10.65 | 0.73 | 1.15 | 0.332 |
| 37 | NTF3 | 13.77 | 1.01 | 9.36 | 0.57 | 1.47 | **0.031** |
| 38 | ANGPT1 | 14.41 | 1.19 | 8.69 | 1.26 | 1.66 | **<0.001** |
| 39 | APLN | 10.31 | 1.54 | 13.53 | 1.40 | -1.31 | 0.106 |
| 40 | BMP2 | 8.55 | 0.60 | 12.36 | 1.20 | -1.45 | **0.002** |
| 41 | SPP1 | 60.66 | 41.33 | 8.06 | 1.73 | 7.52 | **<0.001** |
| 42 | CTSL | 10.79 | 1.08 | 9.82 | 1.10 | 1.10 | 0.569 |
| 43 | C3 | 15.14 | 3.05 | 8.50 | 1.64 | 1.78 | **0.037** |
| 44 | OSTN | 10.13 | 0.86 | 7.10 | 1.17 | 1.43 | 0.220 |
| 45 | ADM2 | 8.04 | 1.00 | 7.60 | 0.93 | 1.06 | 0.541 |
| 46 | STC1 | 9.12 | 1.37 | 8.96 | 1.79 | 1.02 | 0.901 |
| 47 | IL36G | 40.66 | 34.88 | 67.11 | 33.73 | -1.65 | 0.889 |
| 48 | PRLH | 9.01 | 1.96 | 5.98 | 0.94 | 1.51 | 0.246 |
| 49 | IGF1 | 6.51 | 0.64 | 5.54 | 0.79 | 1.17 | 0.433 |
| 50 | IL15 | 6.31 | 0.60 | 4.22 | 0.49 | 1.50 | 0.069 |
| 51 | ADM5 | 2.60 | 0.23 | 14.10 | 2.71 | -5.42 | **<0.001** |
| 52 | NPFF | 4.45 | 0.32 | 7.31 | 0.69 | -1.64 | 0.010 |
| 53 | STC2 | 5.75 | 0.57 | 4.45 | 0.46 | 1.29 | 0.175 |
| 54 | THPO | 5.33 | 0.38 | 4.19 | 0.39 | 1.27 | 0.190 |
| 55 | LEP | 8.10 | 1.98 | 5.39 | 1.39 | 1.50 | 0.155 |
| 56 | IL17C | 6.99 | 0.90 | 4.86 | 0.99 | 1.44 | 0.062 |
| 57 | INHBB | 5.50 | 0.55 | 2.92 | 0.51 | 1.88 | **<0.001** |
| 58 | IL33 | 5.76 | 0.63 | 3.64 | 0.45 | 1.59 | **0.008** |

*Difference in average was tested by a paired two-sample t-test adjusted by false discovery rate.

**Supplemental Table S3**. Information of the eight female common bottlenose dolphins at the start of the experiment.

| Individual # | Age (years) * | Birth experience | Facility |
| --- | --- | --- | --- |
| 1 | 46 | Yes | Adventure World |
| 2 | 21 | Yes |  |
| 3** | 4 | No | Taiji Whale Museum |
| 4 | 2 | No |  |
| 5 | 1 | No |  |
| 6 | 1 | No |  |
| 7 | 33 | Yes | Shimonoseki Marine Science Museum |
| 8 | 5 | No |  |

*Age was estimated by each facility from some published and unpublished sources. For only #8, which was born in captivity, the actual age is shown.

** The individual #3 began to show elevated plasma progesterone concentrations during the seasonal biopsy sampling.
